# Benefits of resistance training are not preserved after cessation of supervised training in prostate cancer patients on androgen deprivation therapy

**DOI:** 10.1002/ejsc.12050

**Published:** 2024-01-30

**Authors:** Lisanne H. P. Houben, Maarten Overkamp, Joan M. G. Senden, Joep G. H. van Roermund, Peter de Vries, Kevin de Laet, Saskia van der Meer, Luc J. C. van Loon, Milou Beelen, Sandra Beijer

**Affiliations:** ^1^ Department of Human Biology NUTRIM School of Nutrition and Translational Research in Metabolism Maastricht University Medical Center + Maastricht The Netherlands; ^2^ Netherlands Comprehensive Cancer Organisation (IKNL) Utrecht The Netherlands; ^3^ TiFN Wageningen The Netherlands; ^4^ Department of Urology Maastricht University Medical Centre + Maastricht The Netherlands; ^5^ Department of Urology Zuyderland Medical Centre Heerlen The Netherlands; ^6^ Department of Urology Máxima Medical Centre Veldhoven The Netherlands; ^7^ Department of Urology Jeroen Bosch Hospital ‘s‐Hertogenbosch The Netherlands

**Keywords:** detraining, hormone therapy, muscle mass, muscle strength, strength training

## Abstract

Resistance exercise training is effective to counteract the adverse effects of androgen deprivation therapy (ADT) on body composition, muscle mass and leg strength in prostate cancer patients (PCa). However, it is unknown whether these effects can be autonomously maintained after cessation of the supervised program. Sixty‐eight PCa patients on ADT were included. The exercise intervention group (EX, *n* = 37) performed 20 weeks of supervised resistance exercise training. Thereafter, patients were advised to autonomously continue exercise training. The control group (CON, *n* = 31) only received usual care. Outcome measures were compared between baseline and after 1 year. Changes during the intervention (baseline vs. 20 weeks) and follow‐up period (20 weeks vs. 1 year) were descriptively explored. In EX, 83% reported to have continued exercise training themselves. After 1 year, fat mass gains were attenuated in EX compared to CON (1.2 ± 2.6 and 2.8 ± 1.9 kg, respectively; time × treatment effect *p* = 0.032). The fat percentage increased, and lean mass and quadriceps muscle cross‐sectional area decreased over time, with no differences between groups (overall 1.6 ± 2.1%, −0.7 ± 2.3 kg and −2.2 ± 2.9 cm^2^, respectively; time effects, all *p* < 0.05). For muscle strength, an increase of ∼5% in EX was observed, significantly different from the ∼10% decrease in CON (*p* < 0.001). Subsequent analyses showed that the initial exercise training‐obtained gains in lean mass, muscle mass and strength in EX compared to CON, declined during the follow‐up period. In conclusion, PCa patients on ADT are not capable to autonomously maintain the exercise‐obtained gains of a 20‐week supervised training program over a subsequent 1‐year period.

## Highlights

- ADT for prostate cancer results in many side effects, including muscle mass loss
- A supervised resistance exercise training program can counteract many side effects
- After cessation of the supervised program, exercise benefits are not effectively preserved
- More focus on sustainability of exercise programs during ADT is required

**Abbreviations**
1RM: one‐repetition maximum
ADT: androgen deprivation therapy
BMI: body mass index
CON: usual care control group
CSA: cross‐sectional area
CT: computed tomography
CWZ: Canisius‐Wilhelmina Hospital
DXA: dual‐energy X‐ray absorptiometry
EX: exercise intervention group
GnRH: gonadotrophin‐releasing hormone
JBZ: Jeroen Bosch Hospital
MMC: Máxima Medical Centre
MUMC+: Maastricht University Medical Centre +
PCa: prostate cancer
SD: standard deviation
T0: experimental test day at baseline
T1: experimental test day after completion of 20 weeks of intervention or usual care
T2: experimental test day 1 year after study enrollment
*η* ^2^_partial_
: partial eta squared

## 1 INTRODUCTION

Androgen deprivation therapy (ADT) is the cornerstone in the treatment of (locally) advanced prostate cancer (PCa) (Cornford et al., 2021; Mottet et al., 2021). About half of the men with PCa will be treated with ADT during their disease trajectory (Meng et al., 2002). By decreasing the androgen concentrations to castration level, ADT inhibits disease progression. However, ADT also causes numerous detrimental side effects, such as a decrease in muscle mass with a concomitant increase in body fat mass (Berruti et al., 2002; Galvão et al., 2008; Smith, 2004). These adverse effects can effectively be counteracted with supervised resistance exercise training. We and others have shown that resistance exercise training increases muscle mass and strength (Dawson et al., 2018; Houben et al., 2023; Newton et al., 2019; Nilsen et al., 2015; Segal et al., 2003; Winters‐Stone et al., 2015a, 2015b), Winters‐Stone, Dieckmann, et al., 2015 and can even reduce fat mass gains in PCa patients treated with ADT (Houben et al., 2023), without additional effect of protein supplementation (Houben et al., 2023).

Although the efficacy of resistance exercise training in PCa patients on ADT is well described, little is known about the sustainability of the improvements in body composition and muscle strength after cessation of the supervised exercise training. Detraining studies in healthy older adults showed that gains in muscle mass and strength obtained during training decline rapidly after complete termination of resistance exercise training (Bickel et al., 2011; Correa et al., 2013, 2016; Ivey et al., 2000; Kalapotha et al., 2010; Kalapotharakos et al., 2007; Lemmer et al., 2000; Trappe et al., 2002). In free‐living situations however, people have the possibility to continue resistance exercise training themselves. Only limited data are available about the sustainability of exercise training‐obtained gains in free‐living situations. Two studies examining the preservation of exercise training‐obtained gains in healthy and chronically diseased older adults, showed that the obtained increases in muscle mass were completely lost and the improvements in strength only partly preserved 1 year after termination of the supervised program (Gylling et al., 2020; Snijders et al., 2019). In the subjects reporting to have autonomously continued resistance exercise training, only an attenuating effect on the declines in muscle mass (Snijders et al., 2019) or in strength, and improvements in waist circumference (Gylling et al., 2020), were observed.

Among PCa patients receiving ADT, data concerning preservation of training‐induced improvements after cessation of a supervised resistance exercise training program are lacking. However, it is important to have insight in the long‐term sustainability of those exercise‐obtained gains, especially as ADT is often prescribed for several years. Therefore, the aim of this study was to assess whether exercise training‐obtained improvements in body composition, muscle mass and muscle strength are maintained after discontinuation of the supervised resistance exercise training program in PCa patients on ADT.

## 2 MATERIALS AND METHODS

### 2.1 Study design

This study was a multicenter partly randomized controlled trial, comparing two exercise intervention groups with a separately recruited usual care control group. The patients recruited for the exercise intervention groups were randomly allocated to 20 weeks of supervised, progressive resistance exercise training with protein supplementation (whey) or with placebo supplementation. Upon completion of the exercise training program, these patients were encouraged to autonomously continue regular exercise training at home or in their neighborhood, but no formal intervention was offered. Patients for the usual care control group were separately recruited. These patients were asked to participate in a study about the effects of ADT, were not informed about the exercise intervention nor encouraged to perform regular exercise, and only received usual care.

An experimental test day was planned at baseline (T0) (before randomization of the intervention groups and immediately after inclusion of the control group), after completion of 20 weeks of intervention or usual care (T1) and 1 year after study enrollment (T2). At the end of T0, patients recruited for the intervention group were randomized over the two intervention groups. The effects of the 20‐week resistance exercise training and protein versus placebo supplementation in PCa patients receiving ADT have been published earlier (Houben et al., 2023).

### 2.2 Patients

Between September 2017 and February 2021, PCa patients were recruited in five hospitals in the southern part of the Netherlands, as previously reported (Houben et al., 2023). Patients were eligible if they started or continued treatment with a gonadotrophin‐releasing hormone (GnRH) agonist or antagonist for at least 6 months. Exclusion criteria were inability to participate in the exercise training regimen, comorbidities that severely compromised physical activity, a high risk of pathological fractures due to bone metastases (as estimated by their treating urologist), an estimated life expectancy <1 year, lactose intolerance or a whey protein allergy, cognitive disorders or severe emotional instability, or unable to speak, understand or read the Dutch language. Potential participants were identified by their treating urologist or urology nurse, and eligible patients were cleared by a sports physician to perform the exercise training program. All patients were provided with the full oral and written study information and signed informed consent before participation. The study was approved by the local Medical Ethical Committee of Maastricht University Medical Centre + (MUMC+), and complied with the principles outlined in the latest version of the Declaration of Helsinki for use of human subjects and tissue. The study was independently monitored by the Clinical Trial Center Maastricht.

### 2.3 Interventional and follow‐up period

The intervention (exercise with protein or placebo supplementation) has been previously described in detail (Houben et al., 2023). In brief, patients in both exercise intervention groups performed an identical, supervised, progressive whole‐body resistance exercise training program (60 min, twice a week) for 20 weeks. The training sessions were performed on strength training equipment and took place in the hospital under direct supervision. Each training session started and ended with 5‐min warm‐up and cooling down on a cycle ergometer. The main program consisted of training on the leg press and leg extension (2 warm‐up sets and 4 working sets, all of 10 repetitions) separated by two upper body exercises (1 warm‐up set and 3 working sets). In cycles of 3 weeks, workload was increased from 65% to 70% one‐repetition maximum (1RM) followed by a reduction in workload to 60% in every 4th week to allow for proper recovery and minimize the risk of injury. After the 4th, 8th, 12th, and 16th week of training, indirect 1RM‐measurements were performed in order to progressively adjust the workload. For patients experiencing medical complications (e.g., treatment‐related issues or injuries), training load was adjusted. In addition, patients in the exercise intervention groups were randomly assigned to ingest a protein or placebo supplement directly after every exercise session and each night before sleep.

For the follow‐up period, patients were advised to continue exercise training with a resistance training component at home or in their neighborhood, and besides being as active as possible. For most patients, this topic was already discussed during the intervention period. In addition, this was explicitly discussed with each patient at the end of the T1 experimental test day. Further information depended on the questions and wishes of the patients. In addition to the oral information, patients were provided with a written document, containing guidelines and tips based on the ‘Dutch Physical Activity Guidelines’.

### 2.4 Outcome measurements

For a detailed description of the assessment of the outcome measurements, we refer to our previous paper (Houben et al., 2023). Below, the main aspects for the outcome parameters are described.

### 2.5 Body composition

Body weight was measured using a digital scale to the nearest 0.1 kg. Height was measured by a fixed stadiometer to the nearest 0.5 cm. Body mass index (BMI) was calculated as kilograms per square meter. Waist circumference was measured at the midpoint between the top of the iliac crest and the lower margin of the lowest palpable rib at the end of a normal expiration. The average of two measurements with ≤1 cm difference was rounded up to the nearest 0.5 cm. Whole body and regional lean mass and fat mass were measured with whole body dual‐energy X‐ray absorptiometry (DXA: Discovery A; Hologic (MUMC+ and Máxima Medical Centre (MMC)) or LUNAR iDXA; GE Healthcare and Horizon A; Hologic (both Jeroen Bosch Hospital (JBZ))). Within‐subject comparisons were only performed over results obtained by the same DXA scan.

### 2.6 Skeletal muscle mass

Skeletal muscle mass was assessed with a single‐slice computed tomography (CT) scan (SOMATOM Definition Flash; Siemens, München, Germany (MUMC + and JBZ) or Ingenuity CT, Philips Medical Systems, Eindhoven, The Netherlands (MMC)) to determine the anatomic cross‐sectional area (CSA) of the quadriceps muscle, as described previously (Backx et al., 2017). A single‐slice image was made 15 cm proximal to the top of the patella of both legs. Quadriceps muscle CSA of the dominant leg was calculated by manual tracing using ImageJ software (version 1.52p, National Institute of Health). All analyses were performed by the same researcher.

### 2.7 Muscle strength

Maximal muscle strength was assessed by 1RM strength tests on the leg press and leg extension machines (Technogym, Milan, Italy). Patients started with a short warm up on a cycle ergometer, after which proper lifting technique was demonstrated and practiced, and a specific warming‐up of 10 and 5 repetitions on ∼50% and 70% of the predicted 1RM was performed. This also served as a practice session to familiarize the participants with the exercise. The 1RM was determined by increasing the load after each successful single lift until failure. A repetition was valid if the entire lift was completed in a controlled manner without assistance.

### 2.8 Habitual physical activity

During the 48 h before all experimental test days, patients were instructed to refrain from any exhaustive physical activity and to arrive at the study location by car or public transportation after an overnight fast. To assess habitual physical activity including possible changes during the course of the study period, patients were instructed to wear a small‐sized triaxial accelerometer (ActiGraph wGT3X‐BT; ActiGraph, Pensacola, FL, USA) on the waist during wakefulness for 7 days prior to the test days. Accelerometer data were analyzed with ActiLife (version 6.13.4; ActiGraph) and average daily step count, percentage of time spent sedentary and in light, moderate, and (very) vigorous activity intensity were calculated. Data were included if patients wore the accelerometer for ≥5 days and ≥10 h per day.

### 2.9 Statistical analyses

Data were expressed as means ± standard deviation (SD) (normally distributed), median and interquartile range (not‐normally distributed), or as frequency and percentages. For all analyses the two intervention groups were merged resulting in one exercise intervention group (EX), as the supplementation was only provided during the intervention period, no additional effect of the protein supplementation was found, and analyzing the two intervention groups separately would result in very small groups.

Baseline characteristics between EX versus the usual care control group (CON) and between patients who completed the 1‐year measurements versus patients loss to follow‐up, were compared using independent samples *t*‐tests (for continuous variables) or chi‐square tests (for categorical variables). As primary analysis, the differences between baseline and 1 year were assessed by a two‐way repeated‐measures ANOVA with time (baseline vs. 1 year) as within subject factor and group (EX vs. CON) as between subject factors. Effect sizes were calculated using partial eta squared (*η* ^2^_partial_). In case of a significant time × treatment interaction, a paired‐sample *t*‐test was performed to detect within group changes over time. For muscle strength, absolute 1RM values could not be compared due to slight differences in leg press and leg extension equipment at the different study locations. Therefore, percentage changes over the 1‐year period were calculated and compared between groups with independent samples *t*‐test.

As secondary, explorative analysis, changes over time during the intervention period (baseline vs. 20 weeks) and follow‐up period (20 weeks vs. 1 year) were described to obtain more insight into the changes during the different periods.

Data were analyzed on an intention‐to‐treat basis. Significance was set at *p <* 0.05. All analyses were performed with the use of IBM SPSS Statistics (version 27.0; IBM Corp.).

## 3 RESULTS

The results of the 20‐week training intervention were reported previously (Houben et al., 2023) and are combined with the 1‐year follow‐up data in this article.

### 3.1 Patients

Ninety‐six patients completed the first 20 weeks of the study. In 68 patients the 1‐year follow‐up measurements were performed. Twenty‐eight patients could not perform the 1‐year follow‐up measurements due to the COVID‐19‐induced lockdown (EX *n* = 16, CON *n* = 2), medical problems (EX *n* = 5, CON *n* = 2) or death (EX *n* = 2, CON *n* = 1).

Baseline characteristics are presented in Table 1. Included patients were on average 71 ± 6 years old, slightly overweight (BMI 27.1 ± 3.4 kg·m^−2^) and treated with ADT for 82 ± 186 days. Compared to patients who completed all measurements, patients loss to follow‐up had less appendicular lean mass, a lower quadriceps CSA, and a lower habitual physical activity level at baseline (*p* ≤ 0.05).

**TABLE 1:** Baseline patients' characteristics.

	One‐year follow‐up completed				Loss to follow‐up		
	Total (*n* = 68)	EX (*n* = 37)	CON (*n* = 31)	*p*‐value^a^	Total (*n* = 28)	EX (*n* = 23)	CON (*n* = 5)
Age (y)	71 ± 6	71 ± 6	71 ± 6	0.909	72 ± 8	73 ± 8	67 ± 10
Time since PCa diagnosis (months)	21 ± 39	18 ± 36	25 ± 42	0.464	30 ± 43	35 ± 46	11 ± 17
Gleason score	8 ± 1	8 ± 1	8 ± 1	0.340	8 ± 1	8 ± 1	9 ± 1
ADT duration (days)	82 ± 186	117 ± 243	40 ± 52	0.068	166 ± 242	199 ± 257	16 ± 5
Bone metastase, *n* (%)	27 (41.5)	11 (32.4)	16 (51.6)	0.116	17 (63.0)	14 (63.6)^b^	3 (60)
Number of comorbidities				0.248			
0	14 (21.2)	5 (13.9)	9 (30.0)		4 (14.3)	3 (13.0)	1 (20.0)
1	22 (33.3)	14 (38.9)	8 (26.7)		11 (39.3)	9 (39.1)	2 (40.0)
≥2	30 (45.5)	17 (47.2)	13 (43.3)		13 (46.4)	11 (47.8)	2 (40.0)
Body weight (kg)	84.0 ± 12.2	84.0 ± 11.3	84.0 ± 13.4	0.998	81.2 ± 12.9	82.3 ± 13.9	76.3 ± 4.8
BMI (kg·m^−2^)	27.1 ± 3.4	27.2 ± 3.0	26.9 ± 3.9	0.645	26.6 ± 3.7	26.9 ± 4.0	25.4 ± 2.2
Fat percentage (%)	30.1 ± 5.6	29.0 ± 4.5	31.4 ± 6.5	0.089	30.9 ± 4.8	30.7 ± 5.0	31.6 ± 4.2
Whole body lean mass (kg)	56.8 ± 6.8	57.8 ± 6.8	55.7 ± 6.8	0.218	53.9 ± 6.7	54.5 ± 7.0	51.0 ± 3.8
Appendicular lean mass (kg)	24.8 ± 3.1	24.9 ± 3.2	24.7 ± 3.1	0.788	23.1 ± 3.2^b^	23.2 ± 3.4	22.5 ± 2.1
Quadriceps muscle CSA (cm^2^)	65.3 ± 9.4	63.6 ± 9.9	67.4 ± 8.4	0.098	59.6 ± 8.8^b^	59.7 ± 8.8	59.0 ± 9.6^b^
Steps per day	6652 ± 2582	6332 ± 2785	7004 ± 2333	0.298	5474 ± 2627^b^	5178 ± 2794	6776 ± 1132
% per day sedentary (%)	75 ± 7	76 ± 7	73 ± 7	0.107	78 ± 7^b^	80±6^b^	71 ± 4

*Note*: Values are means ± SD, or number and (%). Not all data are available for all patients. Data available in ‘total completed group’: time since PCa diagnosis, Gleason score, quadriceps muscle CSA *n* = 67; number of comorbidities *n* = 66; bone metastase, steps per day, % per day sedentary *n* = 65. Data available in ‘completed EX’: number of comorbidities *n* = 36; bone metastase, steps per day, % per day sedentary *n* = 34. Data available in ‘completed CON’: time since PCa diagnosis, Gleason score, number of comorbidities, quadriceps muscle CSA *n* = 30. Data available in ‘total loss to follow up’: bone metastase, quadriceps muscle CSA, steps per day, % per day sedentary *n* = 27. Data available in ‘loss to follow up EX’: bone metastase, quadriceps muscle CSA, steps per day, % per day sedentary *n* = 22.

Abbreviations: ADT, androgen deprivation therapy; BMI, body mass index; CON, usual care control group; CSA, cross‐sectional area; EX, exercise intervention group; PCa, prostate cancer.

^a^ *p*‐value of baseline value of EX compared to CON of patients who completed the 1‐year follow‐up assessments.

^b^ Significantly different from patients in the corresponding group who completed the study (all *p* ≤ 0.05).

At T2, 83% of the patients from EX reported to have autonomously performed exercise training with a resistance‐type component during the follow‐up period. Details are provided in Table 2.

**TABLE 2:** Continuation of exercise training with a resistance‐type component during the follow‐up period.

Continuation of some kind of resistance exercise (yes/no)?	Description
Yes	2x per week for 2 h, combination of RT with other exercise forms, with supervision, only performed for 5 weeks.
Yes	Daily for 45–60 min at home, combination of stretching, ground and RT exercises.
Yes	2x in 3 weeks for 60 min, same RT exercises as during study intervention, at local gym, since end of study intervention.
Yes	2‐3x per week RT for 50–60 min, at local gym without supervision, since end of study intervention till 3 months ago, thereafter daily 20–30 min body weight exercises till 1.5 months ago.
	Other: Endurance sport, 2–3x per week for 30 min, since end of study intervention till 3 months ago; walking, 4x per week 45–60 min, for many years till 3 months ago.
Yes	2x per week for 1 h, same exercises as during study intervention, at local gym, since 3 to 4 weeks after end of study intervention till COVID‐19 induced lockdown (+/− 3 months later); for 3 weeks training restarted.
Yes	Every day 30 min, training with dumb‐bells at home, since end of study intervention till 2 months ago.
	Other: Walking, every day for 1–1.5 h, for many years; cycling on a city bike, 3–4x per week for 1 h, for many years.
Yes	2x per week RT for 45 min, at local gym without supervision, since end of study intervention till COVID‐19 induced lockdown (+/− 3 months later); every day some RT exercise at home.
	Other: Cycling on electric bikes, short distances.
Yes	1x per week for 1 h, combination of RT and AE at physiotherapist, since end of study intervention till COVID‐19 induced lockdown (+/− 3 months later); restarted for 4 weeks.
	Other: Cycling on an electric bike, on average 175 km per week.
Yes	2x per week for 45 min, same exercise/training as during study intervention, at local gym without supervision, since end of study intervention till COVID‐19 induced lockdown (+/− 3 months later).
	Other: Tennis, 3x per week for at least 2 h, since 3–4 years; cycling on electric bike, 3–4x per week for 3 h, for 3–4 years.
Yes	2x per week for 45 min, 1 unsupervised session with same exercises/training as during study intervention plus additional core and stability exercises, 1 supervised session on strength training equipment, at local gym, since end of study intervention.
	Other: Tennis, 2x per week for 1–1.5 h, since many years; cycling on electric bike, 2x per week for 2 h, for many years; endurance sport, 1x per week for 1 h in October–March, for many years.
Yes	2x per week for 1.5 h, same exercise/training as during study intervention, at local gym without supervision, since end of study intervention, last month very low frequency and intensity.
	Other: Golf, 1x per week for 3 h, for 30 years; Endurance sport, 2x per week for 0.5 h, for 8 years.
Yes	3x per week for 1 h, same exercise/training as during study intervention, at local gym, started 1 month ago.
	Other: Walking, every day, with on average ∼40–50 km per week, for >30 years.
Yes	4x per week for 1.5 h, same exercise/training as during study intervention for 0.5 h plus additional walking on treadmill for 1 h, at local gym without supervision, since end of study intervention.
	Other: Walking, almost every day for 2 h, for 2 years; cycling on electric bike, on days with no walking, 2–3 h, for 2 years.
Yes	2 times per week for 1 h, same exercise/training as during study intervention, at local gym without supervision, since end of study intervention.
	Other: Tennis, 3 times per week for 1.5 h, since 27 years; Walking, every day 5 km, for many years; cycling, only during summer period, 50 km per week.
Yes	2x per week for 1 h, same exercise/training as during study intervention, at local gym without supervision, since end of study interventiontill COVID‐19 induced lockdown (+/− 5 months later).
Yes	1x per week for 1 h, same exercise/training as during study intervention without leg press, at local gym without supervision, since end of study intervention till COVID‐19 induced lockdown (+/− 5 months later).
	Other: Cycling on an electric bike, 1x per week 30–45 km, for many years.
Yes	1x per week for 1 h, same exercise/training as during study intervention, under supervision of oncological physiotherapist, since end of study intervention, with break from 2.5 months due to COVID‐19 induced lockdown.
	Other: Walking, 3–4x per week for 1 h and 20 min, for many years.
Yes	2x per week, combination of RT and AE, at physiotherapist, for 1 month.
	Other: Team sport, 1x per week friendly match.
Yes	2x per week RT for 60–75 min, exercise similar as during study intervention plus some additional exercises, at local gym, started 4 months after end of study intervention.
Yes	2x per week for 2 h, RT and cross trainer, training schedule designed by patient himself, at local gym with some supervision, since end of study intervention.
	Other: Walking, every day 1 h, 1x per week 12 km, and 1x per month 30–50 km, for 30 years.
Yes	2x per week RT for 1 h under supervision of physiotherapist, started 3.5 months after end of study intervention.
	Other: Walking, 1x per week max 8 km, started 3.5 months after end of study intervention.
Yes	1–2x per week for 1 h, combination of RT and AE, at local gym, 1x per month supervised session, since end of study intervention.
	Other: Golf, for many years.
Yes	2x per week RT for 60 min, 1x per week balance and AE, 1x per week yoga, all at local gym with supervision, started 1 month after end of study intervention till 2 months ago.
Yes	2x per week 2 h, same exercises as during study intervention, at local unsupervised gym, since end of study intervention.
Yes	5x per week 1.5 h, mainly RT with some AE, at local gym with scare supervision.
	Other: Yoga, 1x per week for 1 h, for 10 years.
No	No exercise continuation.
No	Other: Walking, 2x per week for 1–2 h since 5 weeks, plus 2x per day 1 h, for 7 years.
No	Other: Walking, 2x per day 30 min since many years, plus 1x per week an additional 3 h.
No	Other: Tennis, 2x per week for 75–90 min, for many years; cycling on electric bike, 5x per week for 60 min; walking, every day for 45–60 min.
No	Other: Walking, 1x per day for 30 min, for 40 years.
Unknown	
Unknown	
Unknown	
Unknown	
Unknown	
Unknown	
Unknown	

Abbreviations: AE, aerobic exercise training; RT, resistance exercise training.

### 3.2 Body composition and skeletal muscle mass

Results for body composition and skeletal muscle mass at baseline and after 1 year, are presented in Table 3. For body composition, only results of patients whose T0 and T2 assessment were performed on the same DXA scanner are included. In this subgroup, whole body fat mass and fat percentage were at baseline significantly lower in EX compared to CON (*p* < 0.05). After 1 year, significant differences over time between groups were found for body weight, BMI, waist circumference and whole body fat mass (time × treatment interaction, all *p* < 0.05). Body weight, BMI and waist circumferences increased over time in CON, with no significant changes over time in EX, while fat mass gains were significantly attenuated in EX compared to CON. For fat percentage, whole body lean mass and appendicular lean mass, no significant differences between groups over time were observed. However, in the total population, fat percentage increased, while whole body lean mass and appendicular lean mass decreased over time (1.6 ± 2.1%, −0.7 ± 2.3 kg, −0.5 ± 1.1 kg respectively; time effect, all *p* < 0.05). For quadriceps muscle CSA, no significant baseline differences were found between EX and CON. Over the 1‐year period, quadriceps muscle CSA decreased in the total population (−2.2 ± 2.9 cm^2^, time effect, *p* < 0.001), with no significant differences between groups.

**TABLE 3:** Changes in body composition and muscle mass over time.

	*n*	Baseline	1 year	Time effect	Time × treatment interaction	Within‐group changes over 1 year	
		Mean ± SD	Mean ± SD	*p*‐value	*p*‐value (η^2^_partial_)	Mean ± SD	*p*‐value
Body weight (kg)				<0.001	0.019 (0.086)		
EX	37	84.0 ± 11.3	84.6 ± 10.7			0.6 ± 3.3	0.297
CON	27	85.7±12.7	88.0 ± 13.3			2.4 ± 2.3	<0.001
BMI (kg·cm^−2^)				<0.001	0.020 (0.085)		
EX	37	27.2 ± 3.0	27.4 ± 2.6			0.2 ± 1.1	0.339
CON	27	27.2 ± 3.9	27.9 ± 4.1			0.8 ± 0.7	<0.001
Waist circumference (cm)				0.008	0.022 (0.079)		
EX	37	104.3 ± 10.3	104.5 ± 9.2			0.2 ± 4.4	0.783
CON	29	103.7 ± 10.5	106.4 ± 11.0			2.7 ± 4.1	0.001
Whole body fat mass (kg)^a^				<0.001	0.032 (0.099)		
EX	31	25.4 ± 6.5	26.7 ± 5.3			1.2 ± 2.6	0.011
CON	16	30.9 ± 8.2	33.7 ± 8.9			2.8 ± 1.9	<0.001
Fat percentage (%)^a^				<0.001	0.176 (0.040)		
EX	31	29.3 ± 4.8	30.6 ± 3.5			1.3 ± 2.2	
CON	16	33.9 ± 4.9	36.1 ± 5.0			2.2 ± 1.8	
Whole body lean mass (kg)				0.049	0.950 (<0.001)		
EX	31	57.9 ± 6.7	57.2 ± 6.5			−0.8 ± 2.5	
CON	16	57.7 ± 6.5	57.0 ± 6.5			−0.7 ± 2.0	
Appendicular lean mass (kg)				0.004	0.832 (0.001)		
EX	31	24.9 ± 3.2	24.4 ± 3.1			−0.5 ± 1.2	
CON	16	25.1 ± 2.9	24.5 ± 2.8			−0.6 ± 1.0	
Quadriceps muscle CSA (cm^2^)				<0.001	0.066 (0.054)		
EX	37	63.6 ± 9.9	62.0 ± 9.7			−1.6 ± 2.9	
CON	26	66.8 ± 8.0	63.8 ± 8.3			−3.0 ± 2.9	

*Note*: Values are means ± SD. For DXA measurements, results are only included when the baseline and 1‐year assessment of a patient were performed on the identical scanner.

Abbreviations: BMI, body mass index; CON, usual care control group; CSA, cross‐sectional area; EX, exercise intervention group.

^a^ Significantly different between groups at baseline (*p* < 0.05).

### 3.3 Muscle strength

Over the 1‐year period, muscle strength increased in EX (leg press 4 ± 11%, leg extension 5 ± 16%) and decreased in CON (leg press −10 ± 9%, leg extension −11 ± 13%), resulting in significant differences between groups (both *p* < 0.001).

### 3.4 Habitual physical activity

No baseline differences between groups were found for habitual physical activity. Between baseline and 1 year, average daily step count and percentage of time in moderate activity intensity significantly decreased (−9.7·10^2^±2.2·10^3^ steps and −1.1 ± 2.5% respectively; time effect, both *p* < 0.001), while percentage of time sedentary showed a strong trend toward an increase (1.3 ± 5.6%, time effect, *p* = 0.059), with no differences between groups. No differences between groups or over time were found for percentage time in light and in (very) vigorous activity intensity.

### 3.5 Changes during the intervention and follow‐up period (explorative)

**FIGURE 1:**
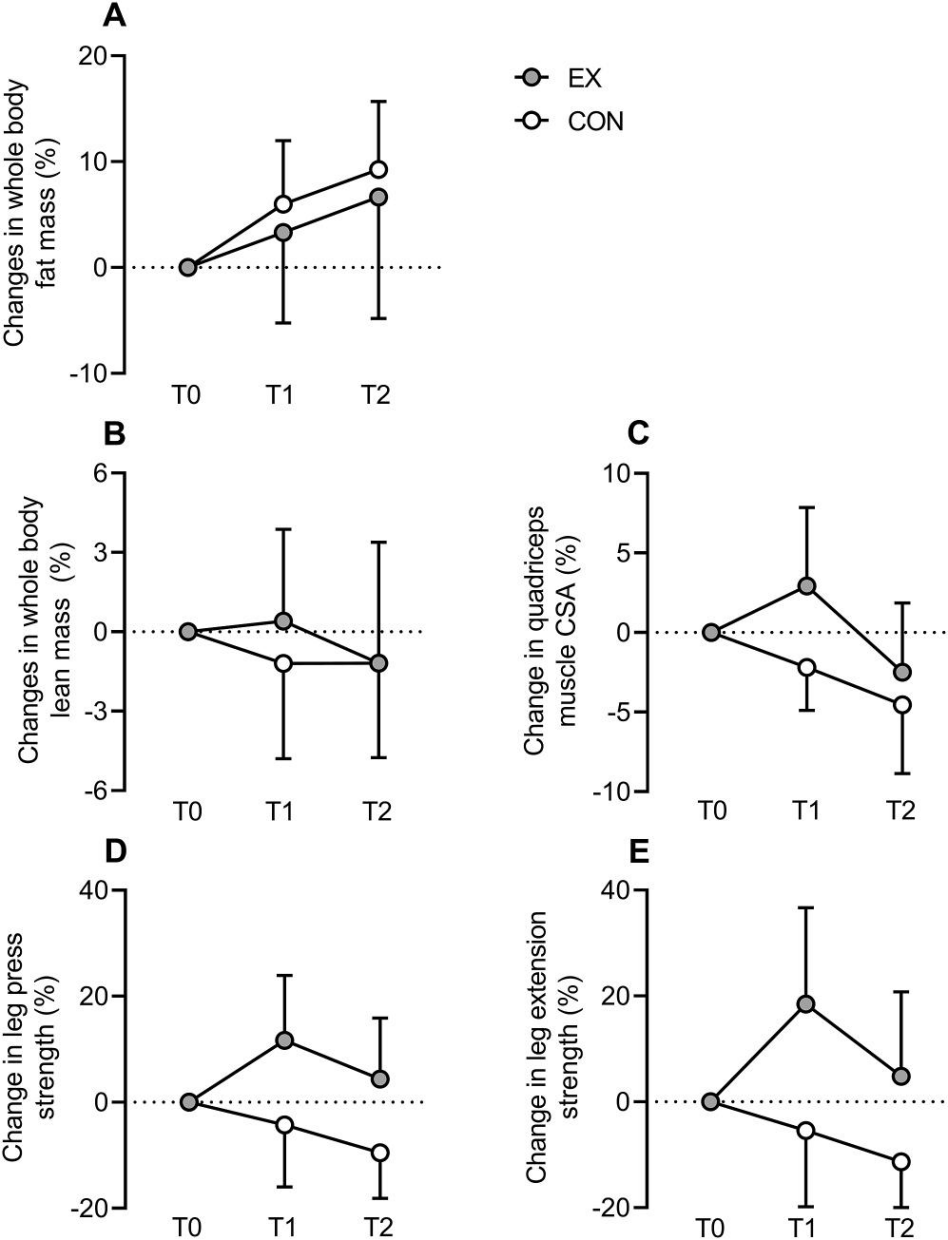
Percentages changes compared to baseline in whole body fat mass (A), whole body lean mass (B), quadriceps muscle cross‐sectional area (C), leg press (D) and leg extension muscle strength (E) after 20 weeks and 1 year. CON, usual care control group; CSA, cross‐sectional area; EX, exercise intervention group; T0, baseline; T1, after 20 weeks; T2, after 1 year.

Figure 1 shows the percentage changes of outcome measures during the intervention and follow‐up period separately. For fat mass, gains were attenuated during the intervention period in EX compared to CON. During the follow‐up period, EX could maintain this obtained benefit, resulting in a still existing between‐group difference after 1 year. Lean mass was maintained and muscle mass increased during the intervention period in EX, while both decreased in CON. However, after cessation of the exercise program, the loss of lean and muscle mass in EX was so large, that after 1 year no between‐group differences existed anymore. In accordance, muscle strength increased in EX during the intervention period, followed by a decrease during the follow‐up period, while in CON a gradual decrease over the entire year was observed. For muscle strength however, there was still a benefit present in EX compared to CON after 1 year.

## 4 DISCUSSION

We have previously shown that a 20‐week supervised resistance exercise training program is effective to combat the adverse effects of ADT in PCa patients. The exercise training program resulted in positive effects on fat mass, muscle mass and muscle strength, compared to usual care only (Houben et al., 2023). In the current study, we assessed whether these beneficial effects are maintained after discontinuation of the supervised intervention. Results showed that 1 year after inclusion—7 months after cessation of the supervised exercise intervention—the exercise training effects were not effectively preserved, though some outcomes were still improved compared to baseline. Fat mass accretion was still attenuated in EX compared to CON, with an accompanying lower increase in waist circumference in EX than in CON. Muscle strength in EX was still ∼5% higher than at baseline, and this significantly differed from CON, in which a ∼10% decline over the 1‐year period was seen. For muscle mass however, no sustained exercise training benefits were observed. Both lean mass as surrogate for muscle mass, and quadriceps muscle CSA declined in EX after cessation of the supervised exercise program to levels lower than at baseline, with no differences between EX and CON after 1 year (Table 2).

Up till now, studies in healthy older adults (Bickel et al., 2011; Correa et al., 2013, 2016; Ivey et al., 2000; Kalapotha et al., 2010; Kalapotharakos et al., 2007; Lemmer et al., 2000; Snijders et al., 2019; Trappe et al., 2002) or older adults with stable chronic diseases (Gylling et al., 2020), provide some insight in the sustainability of gains in muscle mass and muscle strength obtained during an exercise program, showing rapid declines after termination of the intervention. We are the first to explore the long‐term sustainability of the benefits of a supervised exercise program in PCa patients on ADT. Our results revealed that both muscle mass and strength declined following cessation of the supervised exercise program. For muscle strength, these declines were smaller than the initial exercise training‐induced gains, resulting in a small positive net result after 1 year, significantly different from the decline in CON. For muscle mass, however, the declines in the follow‐up period were larger than the initial obtained improvements, resulting in a net decline after 1 year that was no longer different from the decline in CON. This discrepancy between changes in muscle mass and strength seems to be in agreement with previous findings (Bickel et al., 2011; Correa et al., 2013; Gylling et al., 2020; Ivey et al., 2000; Snijders et al., 2019; Trappe et al., 2002), showing a larger decline of muscle mass compared to muscle strength after cessation of an exercise intervention. Probably this is caused by neuromuscular adaptations (Häkkinen et al., 1998) that persist longer than the more temporary gains in muscle tissue mass. For whole body fat mass, the exercise training‐induced attenuation of fat mass gain was maintained during the follow‐up period in EX. Snijders et al. (2019) published one of the few studies assessing the sustained exercise training effects on whole body fat mass as well. However, in their study the exercise training‐induced decline in fat mass was dissipated 1 year after cessation of the supervised intervention.

From a clinical and patient perspective, sustained improvements of muscle strength and attenuation of fat mass gains are relevant profits. Strength is important to independently perform activities of daily living and is associated with survival (Versteeg et al., 2018), while being overweight and obese are associated with the development of multiple comorbidities like cardiovascular diseases (Guh et al., 2009; Koliaki et al., 2019). However, despite a partial preservation of muscle strength after 1 year, substantial decreases in both muscle strength and mass were observed during the follow‐up period. This is remarkable, as our patients had experienced the benefits of training during the intervention period and were strongly encouraged to autonomously continue training. In fact, a large number of our patients (83%) reported to have continued exercise training with a resistance‐type component. Apparently, most PCa patients are not capable of autonomously performing exercise at an intensity sufficient to preserve or further improve exercise training‐induced gains during ADT. This is in agreement with studies in free‐living healthy and older adults with chronic disease (Gylling et al., 2020; Snijders et al., 2019). These studies showed that 1 year after termination of the supervised program, autonomous continuation of resistance exercise only resulted in attenuation of the decline of muscle mass (Snijders et al., 2019) or muscle strength, and improvements in waist circumference (Gylling et al., 2020). Therefore, performing a supervised and structured training program as long as ADT is prescribed (often ≥2 years) would probably be most ideal. Though it seems likely that this would lead to maintenance of training gains, this still needs to be confirmed. Besides physiological factors, other aspects like motivation will determine long‐term efficiency. Furthermore, practical issues, costs, staffing aspects and the extra pressure on the healthcare system should be taken into account as well. Therefore, a broad focus on ways to increase sustainability of exercise regimes is needed, for example, on maintenance training programs, hybrid training regimes (partly autonomously, partly supervised), the use of digital technologies to support home training or educational strategies to teach patients how to exercise effectively and achieve long‐term behavioral changes.

Our study has been hampered by the COVID‐19‐induced lockdown from March 2020 onwards. The follow‐up period of 10 out of 37 (27%) patients in the exercise intervention group fell within this lockdown, considerably restricting the possibilities to continue exercise training. However, a sensitivity analysis without these patients showed similar results. Another limitation is the lack of data concerning additional chemotherapy or radiation therapy during the follow‐up period, as this could possibly have influenced the results.

We conclude that the beneficial effects of a 20‐week supervised resistance exercise training program in PCa patients treated with ADT, are not effectively preserved over a longer time period. One year after the start of the intervention, exercise training benefits are only (partly) preserved for fat mass and muscle strength, but have completely dissipated for muscle mass. Although a high number of patients reported to have continued exercise training, the impact was insufficient to preserve the benefits of the supervised exercise intervention and stress the importance for more guided long‐term strategies. Therefore, in order to offset the detrimental adverse effects of ADT on the long‐term and to maintain body composition and performance benefits, more focus on the sustainability of the effects of exercise programs is required.

## CONFLICT OF INTEREST STATEMENT

LJCvL has received research grants, consulting fees, speaking honoraria, or a combination of these for research on the impact of exercise and nutrition on muscle metabolism, which include funding from companies such as FrieslandCampina and Arla Foods Ingredients. A full overview on research funding is provided at: https://www.maastrichtuniversity.nl/l.vanloon. MB has received a research grant from Arla Foods Ingredients. SB has received a research grant, consulting fees, speaking honoraria, or a combination of these from Baxter, Nutricia and Arla Foods ingredients. None of the other authors has any conflicts of interest to disclose. The results of the study are presented clearly, honestly, and without fabrication, falsification, or inappropriate data manipulation.

## CLINICAL TRIAL REGISTRY NUMBER

This trial is registered at https://trialsearch.who.int/ as NTR 6432 (previously www.trialregister.nl as NL6258).
